# Supplementary material for: Resolving the full spectrum of human genome variation using Linked-Reads
Source: Genome Res. 2019 Apr;29(4):635–45. doi: 10.1101/gr.234443.118 (PMC6442396; doi:10.1101/gr.234443.118)
Supplement: Supplemental Material [file supp_gr.234443.118_Supplemental_longranger-2.2.zip › longranger-2.2.tar.bz2!/lr2.2-public/lib/stan/cmdstan/stan/doc/api/html/check__positive__finite_8hpp_source.html]

Stan: src/stan/math/error\_handling/check\_positive\_finite.hpp Source File


|  |  |
| --- | --- |
|  | Stan  2.5.0  probability, sampling & optimization |


- Main Page
- Related Pages
- Namespaces
- Classes
- Files

- File List
- File Members

All Classes Namespaces Files Functions Variables Typedefs Enumerations Enumerator Friends Macros Pages

- src
- stan
- math
- error\_handling

check\_positive\_finite.hpp

Go to the documentation of this file.

1 #ifndef STAN\_\_MATH\_\_ERROR\_HANDLING\_CHECK\_POSITIVE\_FINITE\_HPP

2 #define STAN\_\_MATH\_\_ERROR\_HANDLING\_CHECK\_POSITIVE\_FINITE\_HPP

3

4 #include <stan/math/error\_handling/check\_positive.hpp>

5 #include <stan/math/error\_handling/check\_finite.hpp>

6

7 namespace stan {

8  namespace math {

9

10  // throws if any element in y is nan

11  template <typename T\_y, typename T\_result>

12  inline bool check\_positive\_finite(const char\* function,

13  const T\_y& y,

14  const char\* name,

15  T\_result\* result) {

16  stan::math::check\_positive(function, y, name, result);

17  stan::math::check\_finite(function, y, name, result);

18

19  return true;

20  }

21

22  }

23 }

24 #endif

check\_positive.hpp

stan::math::check\_positive\_finite

bool check\_positive\_finite(const char \*function, const T\_y &y, const char \*name, T\_result \*result)

**Definition:** check\_positive\_finite.hpp:12

stan::math::check\_finite

bool check\_finite(const char \*function, const T\_y &y, const char \*name, T\_result \*result)

Checks if the variable y is finite.

**Definition:** check\_finite.hpp:52

stan::math::check\_positive

bool check\_positive(const char \*function, const T\_y &y, const char \*name, T\_result \*result)

**Definition:** check\_positive.hpp:54

check\_finite.hpp


---

[ Stan Home Page ]

*© 2011–2014,
Stan Development Team.*
